# Factors associated with repeat contact with an out-of-hours mental health crisis service: an observational study

**DOI:** 10.1136/bmjph-2025-002924

**Published:** 2025-11-10

**Authors:** Kate Gemma Richards, Emily Eyles, Paul Scott, Mark Jackson, Heleni Covary, Theresa Redaniel

**Affiliations:** 1 University of Bristol Medical School, Bristol, UK; 2 Public Health, Bath and North East Somerset Council, Keynsham, UK; 3 National Institute for Health and Care Research Applied Research Collaboration West, Bristol, UK; 4 Bath Mind, Bath, UK; 5 National Cancer Registry, Cork, Ireland

**Keywords:** Mental Health, Public Health, Community Health, Emergencies, Tertiary Prevention

## Abstract

**Introduction:**

Repeat attendances to healthcare services are common. Safe Spaces are innovative non-clinical mental health support services, which aim to address service gaps in crisis management. They are often offered in collaboration with the voluntary and community sector and are delivered in the community as a welcoming and comfortable space for anyone with mental health needs to seek support. To date, there is limited evidence exploring repeat engagement with these services. Our objective was to describe the frequency of repeat contacts with a Safe Space service and to investigate associated factors.

**Methods:**

Data were collected by Bath Mind Breathing Space, in Bath and North East Somerset between April 2020 and June 2022. The primary outcome was repeat contact. Exposure variables included age, gender, ethnicity, existing mental health diagnosis, presenting symptoms and lifestyle and physical health factors. Univariable and multivariable logistic regression analyses were used to assess exposure variables’ association with repeat contact.

**Results:**

Repeat contact with the service occurred in 50% of cases (252). Clients reporting symptoms of depressive disorder (OR 3.29, 95% CI 1.63 to 6.63), or suicidal ideation or self-harm (OR 2.61, 95% CI 1.16 to 5.87) were more likely to have a repeat contact. Having a diagnosis of psychosis (Coeff 3.53, 95% CI 1.67 to 7.39) was associated with an increase in total contacts.

**Conclusions:**

Breathing Space has a high proportion of repeat contacts. Further evaluation is needed to determine how to best support clients with symptoms of depression and suicidal ideation or self-harm or a diagnosis of psychosis to ensure care optimisation.

**WHAT IS ALREADY KNOWN ON THIS TOPIC**

- Mental ill health makes up a substantial cause of disability and, in particular, repeat healthcare service use.

**WHAT THIS STUDY ADDS**

- This observational study looks at unplanned repeat contacts with a novel non-clinical mental health service. This is an area which has not been previously studied.There is a high proportion of unplanned repeat contacts, particularly with clients presenting with symptoms of depressive disorder, suicidal ideation or self-harm.

**HOW THIS STUDY MIGHT AFFECT RESEARCH, PRACTICE OR POLICY**

- Such community mental health services can be designed to ensure that they meet user needs and accommodate repeat contacts.

## Introduction

 In the UK, mental ill health (illness affecting how individuals think, feel or behave which impacts on their ability to function) is the largest cause of disability, accounting for 23% of the burden of disease.^1 2^ People with mental ill health have 3.2 times more emergency department (ED) attendances than those without, and a substantial proportion of repeat ED attendances are among those known to mental health services.^3 4^ A proportion of these additional attendances are for physical health presentations.^3^ However, ED attendance rates for mental ill health rose 133% between 2009/2010 and 2017/2018^4 5^ and have remained at similar levels.^6^ Two-thirds of attendances occur outside of working hours, reflecting difficulty in accessing quality crisis care and pressure on community services.^4 7^ Repeat unplanned attendance increases resource use and affects quality of life.^8^ Furthermore, busy A&E environments may not provide an appropriate calm location to support individuals or may be insufficiently resourced to meet mental health needs.^4^ Assessing factors associated with repeat attendance can help improve care pathways and outcomes.

Safe Spaces are non-clinical community services, which aim to prevent mental health emergencies and address service gaps by providing alternative access to support.^9^ They are supported by the 2019 NHS Mental Health Implementation Plan for England.^10^ Rather than replacing existing crisis teams or mental health services within EDs, Safe Spaces sit alongside these as an alternative form of urgent support. They do not require an appointment and often operate during evenings or weekends. NHS mental health trusts may be involved in provision, but arrangements often involve voluntary and community sector partners, as seen in the Aldershot Safe Haven service.^11^ High numbers of repeat attendances have been identified; for example, the Aldershot service found 97% of contacts to be repeat attendances. However, limited evaluation of these services has been performed, and factors associated with repeat contact were not studied.^11^

Repeat adult mental health presentations to other acute services have been associated with ethnicity, deprivation, existing mental health diagnosis, previous hospital admission and substance misuse.^12^,^15^ For young adults and children, additional factors, including age, gender, reason for presentation and education have also been identified.^16^,^20^ Poor social support and healthcare access have been associated more generally with repeated healthcare use.^21 22^ The ability to generate conclusions from previous studies is limited by discrepancies in primary outcomes and exposure variables studied.^16^,^18^ No studies directly addressed service change. However, suggestions focused on tailored care for these individuals, for which there was some evidence base, focusing resources and considering wider determinants.^13 14 19^

Breathing Space is a non-clinical mental health crisis service, described as a Safe Space. It is provided by Bath Mind and commissioned by Bath & North East Somerset (B&NES), Swindon and Wiltshire Integrated Care Board. The telephone service opened on 13 April 2020 and the face-to-face service on 12 April 2021.

This observational study evaluated contacts with Breathing Space. The aim was to identify the frequency of unplanned repeat contacts and to assess associated factors. This provided the opportunity to analyse repeat contacts in a previously understudied service type. Unplanned contacts reflect felt need, and the analysis of their determinants can help to inform service design and capacity.

## Materials and methods

### Setting

Breathing Space is available to residents over 16 years, living in or registered with a general practitioner in B&NES.^23^ The face-to-face service can be accessed at a community space between 18:00 and 22:30 every day. The telephone service is available between 17:30 and 23:30 Monday to Thursday. Prior to the opening of the face-to-face service, the telephone service provided access 7 days a week. The service is staffed by mental health professionals.

### Participants

Study participants (n=504) were those presenting to the telephone service between 13 April 2020 and 29 June 2022 and the face-to-face service between 12 April 2021 and 29 June 2022. Repeat contacts were excluded if they were a planned follow-up (1299 contacts) as these are not user initiated. No upper time limit was defined for repeat contacts, and no follow-up period was included at the end of recruitment.

### Data source

Data were collected routinely as part of service provision. They were collected separately for each service type by Breathing Space staff consulting with individuals using a computerised system and compiled in an Excel document. Senior staff at ‘Bath Mind’ combined the data from the telephone and face-to-face services into a single set with common user IDs and pseudoanonymised the data. The data was encrypted and sent using the University secure file transfer facility.

### Variables

The primary outcome variable was whether an individual had a repeat contact, defined as more than one contact with the service during the data collection period. This was positive if an individual had more than one contact recorded, either face-to-face or via telephone. Additional outcomes included the total number of contacts.

Exposure variables were selected based on literature review for factors determining repeat attendance to acute healthcare services, with a focus on mental health.^12^,^22^ These were demographic (age, gender, ethnicity), previous mental health history (existing mental health diagnosis), presenting symptom and additional factors (financial concerns, physical health, social isolation and other lifestyle factors, eg, substance misuse). Ethnicity was classified using the UK census categories of white, Asian, black, mixed and other; these were combined into a binary variable (white and Asian/black/mixed/other) due to sample size, which reduced ability to identify subgroup differences.

Existing mental health diagnosis was self-reported and grouped according to the ICD-10 (International Statistical Classification of Diseases and Related Health Problems 10th Revision) mental health diagnosis blocks.^24^ Less common diagnoses (<5 clients) were combined under ‘other’. This group included a mix of organic disorders, disorders due to substance misuse and learning disabilities, which may have different presentations and management. Symptoms on presentation and additional factors were extracted from free text data. These were limited to volunteered information. Current financial concern was recorded as a positive binary outcome (0=none, 1=present) if the individual reported concerns related to money, employment or expenses. Physical health was recorded as positive where a physical health complaint was reported. Social isolation was recorded as present if individuals reported loneliness or isolation. Other lifestyle factors were documented as present if an individual reported current challenges with alcohol, tobacco and/or illicit drugs.

Exposure variables were used to produce a directed acyclic graph to inform analysis (online supplemental figure 1). Deprivation, education and healthcare access were considered, but either insufficient information was routinely collected, or the information was not shared as per the data sharing agreement.

Confounding factors included in the analysis are lifestyle factors, existing mental health diagnosis, symptoms, physical health and social isolation. Age was categorised to facilitate analysis (<25 years, 25–34 years, 35–44 years, 45–54 years, 55–64 years, 65 years and over).

Referral source and presence of an ongoing plan were not considered independent exposure variables and were therefore restricted to descriptive analysis. Definitions for outcome and exposure variables and explanation of category labels are provided in table 1.

**Table 1:** Definitions for outcome and exposure variables and category labels

Variable/category	Definition
Variables	
Age	Age of service user in years at first contact. Calculated using date of birth and date of contact.
Day of week	Day of week of first contact with service.
Ethnicity	Ethnicity collected at any of the first or subsequent contacts. Categorised according to codes used for Mental Health Services Dataset, then reduced to categories used in UK Census and finally to categories of White and other ethnicity to include Asian, Black, mixed and all other.
Existing mental health diagnosis	User reported mental health diagnosis, grouped according to mental health blocks for ICD-10 Version: 2016 (International Statistical Classification of Diseases and Related Health Problems 10th Revision). Collected at first contact.
Financial concerns	Extracted from self-reported free text data on reason for attendance. Binary outcome, recorded as present if individual reported any current financial problem at time of first contact.
First service contact	Type of service used at first contact, either telephone or face-to-face service.
Gender	Gender of user as recorded at any of first or any subsequent contact.
Lifestyle factors	Extracted from self-reported free text data on reason for attendance if individual reported current effect of other lifestyle factor, for example, substance misuse. Reduced to binary outcome.
Number of contacts	Total number of contacts user had with service during period of data collection.
Physical health	Extracted from self-reported free text data on reason for attendance. Binary outcome, recorded as present if individual reported effect of any physical health problem at time of first attendance.
Referral source	Identifies where individual found out about service at time of first contact.
Second service contact	Type of service used at second contact, either telephone or face to face.
Social support/isolation	Extracted from self-reported free text data on reason for attendance. Binary outcome recorded as present if individual reported loneliness or isolation at time of first contact.
Symptoms on presentation	Primary presenting symptoms at first contact with service. Extracted from free text data on reason for attendance.
Category labels	
Behavioural syndrome	Eating disorder, sleep disorder, puerperal disorders.
Bipolar disorder	Bipolar disorder and mania.
Community organisation	Organisation or group run by the community, for example, support group.
Depression and/or anxiety	Either depression, anxiety or both.
Depressive disorder	Depression and depression with anxiety.
Educational organisation	School or higher education institution.
GP	General practitioner or GP service staff.
Loneliness	Report of isolation or loneliness.
Neurotic disorder	Phobias, anxiety disorders, OCD (obsessive-compulsive disorder) or PTSD (post-traumatic stress disorder).
None	No previous diagnosis.
Online	Internet source.
Other (for mental health diagnosis)	Any other including organic disorder, disorder due to substance misuse, learning disabilities.
Other healthcare	Includes secondary care and mental health services including psychological services.
Personality disorder	All personality disorders.
Police	Police including community support officers.
Psychosis (for mental health diagnosis)	Schizophrenia and psychotic disorders.
Psychosis (for symptoms)	Current psychotic symptoms.
Self/family/friend	Any close acquaintance.
Social support service	Service which provides social support, for example, social services, housing services, employment services, care services.
Suicide/self-harm	Ideations or intentions of suicide (act of intentionally carrying out an action to kill oneself) or self-harm (where somebody injures or harms themselves to cope with or express extreme emotional distress and internal turmoil).^31^
third sector	Voluntary or non-profit organisation.

### Statistical analysis

Descriptive analysis was performed, and missing data were reported. For categorical and binary data, percentages are reported. For continuous data, mean and SD are reported. Ongoing plan and referral source were included as the information was pertinent to the service provider.

Univariable logistic regression was performed to assess the binary outcome of repeat contact with the service for pre-identified exposure variables. A multivariable logistic regression model was then constructed including all the exposure and confounding variables. It was not possible to include deprivation, education and healthcare access, as these data were not available. Log-linear models were used to analyse factors associated with the total number of contacts. We have provided exponentiated results to aid interpretation by readers. No upper time limit had been placed for repeat attendances and therefore a sensitivity analysis was performed with the follow-up truncated at 90 days, 180 days and 365 days (based on a median time to second contact of 7 days).

Imputation models were derived for each missing variable and included: the exposures of interest, outcomes and all other variables with or without missing data. 20 complete datasets were constructed and the results were combined using Rubin’s Rules.^25^ The results of the regression analysis were based on the imputed dataset. It was not possible to impute ethnicity due to the large amount of missing data and perfect prediction. Data management and statistical analysis was performed using Stata V.17.

### Patient and public involvement

Patients or the public were not involved in the design, conduct or reporting of this work.

## Results

### Descriptive analysis

A total of 504 individuals contacted the services, with an average of 2084 contacts per annum to the telephone service and 2040 contacts per annum to the face-to-face service. For 443 individuals, their first contact was with the telephone service (table 2).

**Table 2:** Presenting characteristics of users of Breathing Space service, April 2020–June 2022

Variable	All users	Single attendance	Repeat attendance
	N	N (%)	N (%)
First service contact			
Phone	443	225 (50.8)	218 (49.2)
Face to face	61	27 (44.3)	34 (55.7)
Referral source			
3rd sector	76	36 (47.4)	40 (52.6)
Community or educational organisation	106	48 (45.3)	58 (54.7)
GP	40	17 (42.5)	23 (57.5)
Other healthcare	55	29 (52.7)	26 (47.3)
Online	23	11 (50.0)	11 (50.0)
Self/family/friend	35	15 (42.9)	20 (57.1)
Police, social support service or other	30	13 (43.3)	17 (56.7)
(missing)	139	83	56
Ongoing plan			
No	16	7 (43.8)	9 (56.3)
Yes	410	195 (47.6)	215 (52.4)
(missing)	78	50	28

GP, general practitioner.

Half of service users had more than one contact. Of those whose first contact was to the telephone service, 49.2% (n=218/443) had a second contact, compared with 55.7% (n=34/61 clients) for the face-to-face service (table 2). Contacts were higher on weekdays, with only 4.4% (22/504) of first contacts on a Sunday (table 2). The mean age of clients was 44.3 years (SD 17.3). Half (54.3%, n=165/304) of female clients had repeat contact, compared with 47.4% of males (n=83/175) (table 3).

**Table 3:** Sociodemographic and presenting characteristics of users of service by outcomes, April 2020–June 2022

Variable	All users	Single attendance	Repeat attendance	Number of contacts
	N	N (%)	N (%)	Median (IQR)
Sociodemographic factors				
Age				
<25 years	74	33 (44.6)	41 (55.4)	2.5 (5)
≥25 and <35 years	74	36 (48.6)	38 (51.4)	2 (3)
≥35 and <45 years	55	22 (40.0)	33 (60.0)	2 (3)
≥45 and <55 years	94	45 (47.9)	49 (52.1)	2 (9)
≥55 and <65 years	62	27 (43.5)	35 (56.5)	2 (3)
≥65 years	57	21 (36.8)	36 (63.2)	2 (5)
(missing)	88	68	20	1 (0)
Mean (SD)	44.3 (17.3)	43.7 (17.4)	44.7 (17.3)	
Gender				
Female	304	139 (45.7)	165 (54.3)	2 (4)
Male	175	92 (52.6)	83 (47.4)	1 (3)
Non-binary/transgender	5			1 (17)
(missing)	20	18	2	1 (0)
Ethnicity				
White	334	133 (39.8)	201 (60.2)	2 (6)
Black, Asian, mixed or other	25	7 (28.0)	18 (72.0)	4 (7)
(missing)	145	112	33	1 (0)
Mental or physical health problems				
Existing mental health diagnosis				
None reported or missing	164	100 (61.0)	64 (39.0)	1 (0)
Bipolar disorder	31	11 (35.5)	20 (64.5)	3 (9)
Depressive disorder (including depressive and anxiety)	173	76 (43.9)	97 (56.1)	2 (4)
Neurotic disorder	80	41 (51.3)	39 (48.8)	1 (3)
Personality disorder	17	10 (58.8)	7 (41.2)	8 (93)
Psychosis	15	5 (33.3)	10 (66.7)	2.5 (11)
Other	24	9 (37.5)	15 (62.5)	1 (2)
Symptoms on presentation				
None reported	61	41 (67.2)	20 (32.8)	1 (1)
Depression and/or anxiety	179	69 (38.5)	110 (61.5)	2 (5)
Loneliness	42	25 (59.5)	17 (40.5)	1 (3)
Suicidal thoughts/self-harm	67	29 (43.3)	38 (56.7)	2 (7)
Psychosis or other	155	88 (56.8)	67 (43.2)	1 (3)
Financial concerns				
None	424	202 (47.6)	222 (52.4)	2 (4)
Yes	19	8 (42.1)	11 (57.9)	3 (3)
(missing)	61	42	19	1 (1)
Lifestyle factors				
None	415	197 (47.5)	218 (52.5)	2 (4)
Yes	27	12 (44.4)	15 (55.6)	3 (7)
(missing)	62	43	19	1 (1)
Physical health				
Not affected	396	191 (48.2)	205 (51.8)	2 (4)
Affected	45	18 (40.0)	27 (60.0)	2 (5)
(missing)	63	43	20	1 (1)
Social support/isolation				
No concerns	340	167 (49.1)	173 (50.9)	1 (3)
Isolation	102	42 (41.2)	60 (58.8)	2 (5)
(missing)	62	43	19	1 (1)

Those with no mental health diagnosis were more likely to use the service once (61.0%, n=100/164). among those with a mental health diagnosis, those with bipolar disorder (64.5%, n=20/31), depressive disorders (56.1%, n=97/173), psychotic disorders (66.7%, n=10/15) and other diagnoses (includes organic disorders, disorders due to substance misuse and learning disabilities) (62.5%, n=15/24) had higher repeat attendances (table 3).

Depression and/or anxiety were the most reported symptoms (in 35.5%, n=179/504). Repeat contact was common in this group (61.5%, n=110/179) and in those with thoughts of suicide or self-harm (56.7%, n=38/67) (table 3).

### Regression analysis

Symptoms of depression and/or anxiety (OR 3.29, 95% CI 1.63 to 6.63) and of thoughts of suicide or self-harm (OR 2.61, 95% CI 1.61 to 5.87) were positively associated with increased odds of repeat contact. Having an existing mental health diagnosis of less common diseases (combined as ‘other’) was associated with increased odds of repeat contact in univariable and multivariable regression (OR 3.32, 95% CI1.14 to 9.73) (table 4). This estimate is imprecise and, due to the heterogeneity of this category, the result is difficult to interpret. Both these findings persisted in the sensitivity analysis (online supplemental table 1).

**Table 4:** Association of user sociodemographic characteristics and clinical presentation with repeat attendance

Variable	All users	Repeat attendance				Total number of contacts (log exponentiated)			
		Univariable		Multivariable		Univariable		Multivariable	
	N	OR	95% CI	OR	95% CI	Coeff	95% CI	Coeff	95% CI
Age									
<25 years	74	Ref		Ref		Ref			
≥25 and <35 years	74	0.84	0.43 to 1.62	1.02	0.51 to 2.07	0.75	0.48 to 1.17	0.81	0.52 to 1.26
≥35 and <45 years	55	0.98	0.49 to 1.92	1.28	0.60 to 2.71	0.71	0.46 to 1.12	0.83	0.52 to 1.36
≥45 and <55 years	94	0.84	0.45 to 1.55	0.92	0.47 to 1.83	0.90	0.59 to 1.36	0.93	0.61 to 1.43
≥55 and <65 years	62	0.95	0.47 to 1.91	1.05	0.49 to 2.22	0.72	0.45 to 1.15	0.77	0.49 to 1.22
≥65 years	57	1.20	0.59 to 2.41	1.55	0.69 to 3.48	0.91	0.57 to 1.45	1.02	0.62 to 1.68
Gender									
Female	304	Ref		Ref		Ref		Ref	
Male	175	0.77	0.53 to 1.11	0.76	0.51 to 1.14	0.79	0.62 to 1.03	0.84	0.64 to 1.07
Non-binary/transgender	5	0.55	0.09 to 3.41	0.49	0.07 to 3.34	1.13	0.34 to 3.74	0.91	0.28 to 2.97
Ethnicity									
White	334	Ref		Ref		Ref		Ref	
Asian/black/mixed/other	25	1.78	0.75 to 4.27	1.80	0.68 to 4.71	1.27	0.75 to 2.18	1.13	0.66 to 1.92
Mental or physical health problems									
Existing mental health diagnosis									
None reported	164	Ref		Ref		Ref		Ref	
Bipolar disorder	31	2.81	1.26 to 6.26	1.95	0.82 to 4.59	2.12	1.26 to 3.60	1.67	0.96 to 2.86
Depressive disorder (including depression and anxiety)	173	1.97	1.28 to 3.05	1.39	0.86 to 2.25	1.34	0.99 to 1.79	1.03	0.75 to 1.40
Neurotic disorder	80	1.47	0.86 to 2.52	1.18	0.65 to 2.12	1.13	0.79 to 1.63	0.94	0.64 to 1.39
Personality disorder	17	1.08	0.39 to 2.99	0.94	0.31 to 2.77	1.21	0.61 to 2.39	1.09	0.55 to 2.20
Psychosis	15	3.09	1.01 to 9.47	2.70	0.80 to 9.12	4.14	2.01 to 8.50	3.53	1.67 to 7.39
Other	24	2.58	1.06 to 6.24	3.32	1.14 to 9.73	2.10	1.16 to 3.74	2.61	1.36 to 5.00
Symptoms on presentation									
None reported	61	Ref		Ref		Ref		Ref	
Depression and/or anxiety	179	3.27	1.77 to 6.04	3.29	1.63 to 6.63	2.23	1.51 to 3.32	2.20	1.43 to 3.39
Loneliness	42	1.39	0.62 to 3.15	1.05	0.41 to 2.64	1.51	0.88 to 2.56	1.22	0.68 to 2.18
Psychosis	10	1.37	0.35 to 5.40	0.73	0.15 to 5.87	1.08	0.44 to 2.69	0.54	0.20 to 1.49
Suicidal thoughts/self-harm	67	2.67	1.40 to 5.99	2.61	1.16 to 5.87	2.32	1.45 to 3.71	1.99	1.20 to 3.32
Other	145	1.58	0.85 to 3.02	1.76	0.86 to 3.58	1.39	1.08 to 2.10	1.46	0.94 to 2.27
Financial concerns									
None	424	Ref		Ref		Ref		Ref	
Yes	19	1.22	0.49 to 3.05	1.60	0.62 to 4.16	1.13	0.59 to 2.14	1.36	0.73 to 2.53
(missing)	61								
Lifestyle factors									
None	415	Ref		Ref		Ref		Ref	
Yes	27	1.16	0.54 to 2.51	1.26	0.55 to 2.86	1.26	0.75 to 2.14	1.32	0.79 to 2.23
(missing)	62								
Physical health									
Not affected	396	Ref		Ref		Ref		Ref	
Affected	45	1.37	0.74 to 2.57	1.22	0.61 to 2.43	1.20	0.79 to 1.82	1.09	0.73 to 1.65
(missing)	63								
Social support/isolation									
No concerns	340	Ref		Ref		Ref		Ref	
Isolation	102	1.25	0.81 to 1.96	1.47	0.81 to 2.66	1.21	0.90 to 1.63	1.32	0.94 to 1.86
(missing)	62								
First service contact									
Phone	443	Ref		Ref		Ref		Ref	
Face to face	61	1.30	0.76 to 2.23	1.28	0.69 to 2.38	1.13	0.78 to 1.63	1.07	0.72 to 1.58

Having a diagnosis of psychotic disorder increased the number of contacts more than three-fold (Coeff 3.53, 95% CI 1.67 to 7.39), but was not associated with repeat attendance on multivariable regression analysis. Presenting with symptoms of depression and/or anxiety and of thoughts of suicide or self-harm increased the number of contacts twofold (Coeff 2.20, 95% CI 1.43 to 3.39 and Coeff 1.99, 95% CI 1.20 to 3.32, respectively). Having an existing mental health diagnosis of less common diseases also increased the number of contacts two-fold (Coefficient 2.61, 95% CI 1.36 to 5.00). No other factor was associated with repeat or increased number of contacts (table 4). These findings were present throughout the sensitivity analysis.

When the follow-up period was truncated at 90 days those with an existing diagnosis of bipolar disorder had increased odds of repeat contact (OR 2.44, 95% CI 1.02 to 5.86) and had an increased number of contacts (Coeff 1.92, 95% CI 1.12 to 3.28) (online supplemental table 1). These findings did not persist over longer durations of follow-up.

## Discussion

### Main findings of this study

Repeat contact rates were high, accounting for 93% of contacts, with 50% of service users having more than one contact. Depression and/or anxiety were the most reported symptoms, and together with symptoms of suicide or self-harm, were associated with repeat contact and number of contacts. Psychotic disorder was positively associated with the number of contacts.

### What is already known on this topic

Repeated contact is comparable to that seen in the evaluation of a Safe Haven in Aldershot, accounting for 93% compared with 97% of contacts.^11^ Cullen *et al* recorded repeat attendance rates to ED at 25%,^20^ while Lunawat and Karale reported rates for a Crisis team of 10.4% for 1 year.^14^ Higher repeat contact rates may be expected in mental health services as mental ill health is a risk factor for repeat attendance.^21 22 26 27^ It may also reflect the accessibility of Safe Spaces, preferences for non-clinical services, or the availability of other services. Safe Spaces may attract those with no formal mental health diagnosis who have less access to follow-up. No evidence was found for a difference in repeat contacts between service types, where a service is provided in both a face-to-face and telephone format.

The proportion of females attending was consistent with the findings of Lunawat and Karale^14^ and supports current literature that men make fewer visits to mental health professionals and in particular to general care, preferring to seek specialist care and medication.^28^ This may be an important consideration when planning the services to ensure equitable access. This pattern is not universally reported and was not seen in Beck *et al*’s review of ED attendances.^15^

The prevalence of depressive disorder and symptoms of depression among service users reflects mixed anxiety and depression being the most common mental health disorder in the UK.^29^ High rates of symptoms of suicide and self-harm in repeat service users are consistent with findings by Cullen *et al*.^20^ Current symptoms of depression and/or anxiety, separate to an existing mental health diagnosis, are not identified in the literature as being associated with repeat attendance. They do not feature at all in Kromka and Simpson’s review of mental health ED return visits.^17^ This discrepancy may reflect a lack of focus on presenting symptoms in previous studies. It may also be due to Breathing Space being a non-clinical service and therefore attracting those with symptoms but no formal diagnosis.

Having an existing mental health diagnosis is associated with increased contacts.^12^,^14^ In the review by Kromka and Simpson, the presence of psychotic and personality disorders is investigated in frequent users.^13^ An association was demonstrated for personality disorder in 63% of studies and for schizophrenia in 82% of studies, although this decreased to 33% of studies for other psychotic disorders. Beck *et al* and Kromka and Simpson found existing diagnoses of substance misuse-related disorders and learning disability to be associated with increased service use.^13 15^ The literature is broadly reflected in our findings, indicating that the service is not directing clients to alternative care more frequently or continues to remain beneficial.

### What this study adds

This study analysed a new non-clinical service design in a local setting. This study supports findings by a similar service in Aldershot.^11^ High rates of repeat contact with Safe Space services compared with current literature may be associated with ease of access, greater acceptability of repeatedly accessing a non-clinical service or limited access to alternative follow-up.

The study expands the conversation around repeat contacts and highlights interactions between diagnoses or symptoms of mental ill health and repeat contact. In doing so, it identifies who uses this kind of service and how, and more importantly, underscores where additional support might be needed, in terms of staff training, resources or links to other services. For example, an increased odds of repeat attendance in those presenting with depression or anxiety symptoms indicates a need to develop resources specific to this group. This will assist in the design and implementation of similar services as they are rolled out across England.

The study replicates many of the findings of previous studies looking at repeat attendance, with additional insights. Psychotic disorders were correlated with number of contacts even after adjusting for confounding, confirming findings of an association between these disorders and frequent contact. No significant association was found with repeat contact; however, the wide CI suggests this may have been limited by precision. While confirming that existing mental health diagnoses are common among service users, there is also a significant proportion of users who do not have a mental health diagnosis. This highlights the potential of Safe Spaces to address population needs not covered by traditional systems. Previous studies have highlighted the relevance of alternative interventions in supporting mental health in particular groups.^30^

Together, these findings can assist in understanding levels of need in different patient groups and can assist similar services in their design and in planning to meet this need.

### Strengths and limitations of this study

The main advantage of the study is the use of a client dataset which collected prospective real-time information using a standardised tool, limiting information bias. Recall bias was minimised by collecting contemporaneous information and observer bias by the interviewer being blinded to repeat contact. Information collected included sociodemographic factors, as well as clinical data.

However, the use of the dataset presented its own limitations. Lifestyle and physical health factors could only be extracted from free text, possibly causing under-reporting. This could have caused differential bias between repeat and non-repeat users, overestimating observed findings. Nevertheless, we did not find any associations between lifestyle and physical health factors and our outcomes; hence, the effect of the bias is minimal. The routine data source did not include information on deprivation, education or healthcare access and the information required to generate this was not shared as part of the data sharing agreement. This limits the ability to identify structural factors such as socioeconomic status that may be associated with repeat attendance. Data were also not collected on support received during the first contact, which could have influenced repeat attendance.

Furthermore, there was a large amount of missing data, particularly for ethnicity (28.77%) and age (17.5%). This could be due to reluctance to disclose data, time pressures or data collection tools. While it is not possible to know if these data were missing at random, missingness is not associated with the outcome and therefore bias is minimised. Due to sample size, we were not able to review ethnicity by subgroup, which may obscure important differences.

The limited sample size affected the precision of the results and therefore the ability to make conclusions about the full range of factors that may affect repeat use. Nevertheless, we have observed associations beyond what is considered chance.

Data collection processes resulted in limitations. No selection took place as all user-initiated contacts were included. This limits control over selection bias and may affect generalisability. Selection bias may have arisen as the service is provided in English, which may exclude those who do not speak English or dissuade them from repeat use. However, analysis was consistent with local population ethnicity.

The mean time between the initial and repeat attendance was 49.99 days (SD 97.78 days). Therefore, stopping data collection without a follow-up period for any repeat contacts may have resulted in underestimation of repeat users or number of contacts. No upper time limit was set for repeat contact and therefore those contacting the service earlier in the data collection period may have been more likely to represent. These are common challenges in evaluating repeat presentation.^7 15^ Individuals may also have been prevented from repeated use of the service due to moving out of area. We were unable to collect any data about this, as data was only collected for contacts with the service.

This study only reviewed a single service and population group. The findings are applicable in the planning of similar services. However, the local nature of the service and small sample size would place some restriction on generalisability. Application to paediatric populations is limited.

### Implications and conclusions

These findings can inform the ongoing work by Breathing Space and similar services. Focus should be placed on identifying users with these risk factors and providing increased signposting and assistance to access care, treatment and support that can meet their needs. This will require strong links with other services.

These findings and further analysis could inform service design, staff training and planning of services; for example, confirming benefits of two access options in reaching diverse individuals. Qualitative research in this area would provide additional insight.

An assumption is often made that repeat attendance to healthcare services is a negative outcome. While there is an associated burden and cost, consideration must be made as to whether some degree of repeat attendance is required to meet mental health needs, and in fact demonstrates the acceptability and value. There may also be a reduction in pressure on NHS services. Unfortunately, it was not possible to link with NHS data during this study due to information governance limitations. This would therefore require further research to measure.

## Supplementary material

10.1136/bmjph-2025-002924 online supplemental table 1

10.1136/bmjph-2025-002924 online supplemental figure 1

## Data Availability

Data may be obtained from a third party and are not publicly available.
