# Supplementary material for: Factors associated with repeat contact with an out-of-hours mental health crisis service: an observational study
Source: BMJ Public Health. 2025 Nov 10;3(2):e002924. doi: 10.1136/bmjph-2025-002924 (PMC12606496; doi:10.1136/bmjph-2025-002924)
Supplement: online supplemental table 1 [file bmjph-3-2-s001.docx]

|  | **Truncated at <=90 days (n=464)** | | | | | | | **Truncated at <=180days (n=480)** | | | | | | | **Truncated at <=365days (n=499)** | | | | | | |
| --- | --- | --- | --- | --- | --- | --- | --- | --- | --- | --- | --- | --- | --- | --- | --- | --- | --- | --- | --- | --- | --- |
| **Variable** | **All users** | **Repeat Attendance** | | **Total number of calls (log)** | | **Total number of calls (log, Exponentiated)** | | **All users** | **Repeat Attendance** | | **Total number of calls (log)** | | **Total number of calls (log, Exponentiated)** | | **All users** | **Repeat Attendance** | | **Total number of calls (log)** | | **Total number of calls (log, Exponentiated)** | |
|  |  | **Multivariable** | | **Multivariable** | | **Multivariable** | |  | **Multivariable** | | **Multivariable** | | **Multivariable** | |  | **Multivariable** | | **Multivariable** | | **Multivariable** | |
|  | **N** | **OR** | **95% CI** | **Coeff** | **95% CI** | **Coeff** | **95% CI** | **N** | **OR** | **95% CI** | **Coeff** | **95% CI** | **Coeff** | **95% CI** | **N** | **OR** | **95% CI** | **Coeff** | **95% CI** | **Coeff** | **95% CI** |
| **Socio, demographic factors** | | | | | | | | | | | | | | | | | | | | | |
| **Age** | | | |  |  |  |  |  |  |  |  |  |  |  |  |  |  |  |  |  |  |
| **<25 years** | 67 | Ref |  |  |  |  |  | 74 | Ref |  |  |  |  |  | 74 | Ref |  |  |  |  |  |
| **>=25 & <35 years** | 67 | 1.04 | 0.49, 2.19 | -0.19 | -0.62,  0.25 | 0.83 | 0.54,  1.29 | 69 | 0.89 | 0.43,  1.82 | -0.28 | -0.72,  0.15 | 0.75 | 0.49,  1.16 | 74 | 1.03 | 0.51,  2.07 | -0.20 | -0.64,  0.23 | 0.82 | 0.53,  1.26 |
| **>=35 & <45 years** | 53 | 1.48 | 0.69, 3.19 | -0.04 | -0.50,  0.42 | 0.96 | 0.60,  1.52 | 53 | 1.21 | 0.57,  2.55 | -0.14 | -0.60,  0.32 | 0.87 | 0.55,  1.38 | 53 | 1.19 | 0.57,  2.50 | -0.17 | -0.64,  0.30 | 0.84 | 0.53,  1.34 |
| **>=45 & <55 years** | 86 | 0.98 | 0.48, 1.99 | 0.00 | -0.43,  0.43 | 1.00 | 0.65,  1.53 | 89 | 0.85 | 0.43,  1.68 | -0.07 | -0.49,  0.36 | 0.94 | 0.61,  1.43 | 93 | 0.91 | 0.46,  1.80 | -0.05 | -0.48,  0.38 | 0.95 | 0.62,  1.46 |
| **>=55 & <65 years** | 53 | 0.99 | 0.44, 2.22 | -0.28 | -0.75,  0.19 | 0.76 | 0.47,  1.21 | 56 | 0.87 | 0.40,  1.90 | -0.28 | -0.75,  0.19 | 0.76 | 0.47,  1.20 | 61 | 1.01 | 0.47,  2.13 | -0.25 | -0.71,  0.21 | 0.78 | 0.49,  1.24 |
| **>=65 years** | 54 | 1.85 | 0.78, 4.39 | 0.18 | -0.32,  0.67 | 1.19 | 0.73,  1.96 | 54 | 1.47 | 0.63,  3.43 | 0.08 | -0.42,  0.57 | 1.08 | 0.66,  1.78 | 57 | 1.52 | 0.66,  3.49 | 0.02 | -0.47,  0.52 | 1.02 | 0.63,  1.68 |
|  |  |  |  |  |  |  |  |  |  |  |  |  |  |  |  |  |  |  |  |  |  |
| **Gender** | | | |  |  |  |  |  |  |  |  |  |  |  |  |  |  |  |  |  |  |
| **Female** | 277 | Ref |  | Ref |  | Ref |  | 288 | Ref |  | Ref |  | Ref |  | 300 | Ref |  | Ref |  | Ref |  |
| **Male** | 162 | 0.75 | 0.49, 1.16 | -0.29 | -0.55,  -0.03 | 0.75 | 0.58,  0.97 | 167 | 0.76 | 0.50,  1.16 | -0.25 | -0.51,  0.01 | 0.78 | 0.60,  1.01 | 174 | 0.77 | 0.51,  1.15 | -0.20 | -0.45,  0.06 | 0.82 | 0.63,  1.06 |
| **Non binary/Transgender** | 5 | 0.63 | 0.09, 4.43 | 0.06 | -1.10,  1.22 | 1.06 | 0.33,  3.39 | 5 | 0.51 | 0.07,  3.54 | -0.04 | -1.22,  1.14 | 0.96 | 0.30,  3.13 | 5 | 0.48 | 0.07,  3.30 | -0.08 | -1.27,  1.11 | 0.92 | 0.28,  3.04 |
|  |  |  |  |  |  |  |  |  |  |  |  |  |  |  |  |  |  |  |  |  |  |
| **Ethnicity** | | | |  |  |  |  |  |  |  |  |  |  |  |  |  |  |  |  |  |  |
| **White** | 299 | Ref |  | Ref |  | Ref |  | 312 | Ref |  | Ref |  | Ref |  | 329 | Ref |  | Ref |  | Ref |  |
| **Asian/Black/Mixed/Other** | 24 | 1.97 | 0.73, 5.36 | 0.12 | -0.40,  0.65 | 1.13 | 0.67,  1.91 | 25 | 1.95 | 0.73,  5.20 | 0.14 | -0.39,  0.66 | 1.14 | 0.68,  1.93 | 25 | 1.82 | 0.69,  4.80 | 0.11 | -0.42,  0.64 | 1.12 | 0.66,  1.90 |
|  |  |  |  |  |  |  |  |  |  |  |  |  |  |  |  |  |  |  |  |  |  |
| **Mental or physical health problems** | | | | | | | | | | | | | | | | | | | | | |
| **Existing mental health diagnosis** | | | |  |  |  |  |  |  |  |  |  |  |  |  |  |  |  |  |  |  |
| **None reported** | 152 | Ref |  | Ref |  | Ref |  | 156 | Ref |  | Ref |  | Ref |  | 162 | Ref |  | Ref |  | Ref |  |
| **Bipolar disorder** | 31 | 2.44 | 1.02, 5.86 | 0.65 | 0.12,  1.19 | 1.92 | 1.12,  3.28 | 31 | 2.15 | 0.90,  5.13 | 0.54 | 0.00,  1.09 | 1.72 | 1.00,  2.97 | 31 | 1.96 | 0.83,  4.62 | 0.50 | -0.04,  1.05 | 1.65 | 0.96,  2.86 |
| **Depressive disorder (including depressive & anxiety)** | 153 | 1.31 | 0.78, 2.21 | 0.10 | -0.22,  0.43 | 1.11 | 0.80,  1.53 | 159 | 1.28 | 0.77,  2.13 | 0.04 | -0.29,  0.36 | 1.04 | 0.75,  1.43 | 170 | 1.35 | 0.83,  2.20 | 0.04 | -0.28,  0.36 | 1.04 | 0.76,  1.43 |
| **Neurotic disorder** | 72 | 1.13 | 0.60, 2.14 | 0.01 | -0.38,  0.40 | 1.01 | 0.68,  1.49 | 77 | 1.16 | 0.63,  2.14 | -0.03 | -0.41,  0.36 | 0.97 | 0.66,  1.44 | 79 | 1.14 | 0.63,  2.07 | -0.06 | -0.44,  0.33 | 0.95 | 0.64,  1.39 |
| **Personality disorder** | 17 | 1.10 | 0.37, 3.33 | 0.22 | -0.46,  0.89 | 1.24 | 0.63,  2.44 | 17 | 1.02 | 0.34,  3.07 | 0.16 | -0.53,  0.85 | 1.17 | 0.59,  2.33 | 17 | 0.93 | 0.31,  2.78 | 0.09 | -0.60,  0.79 | 1.10 | 0.55,  2.20 |
| **Psychosis** | 14 | 2.93 | 0.83, 10.33 | 1.33 | 0.58,  2.09 | 3.79 | 1.78,  8.07 | 15 | 2.97 | 0.87,  10.13 | 1.31 | 0.56,  2.05 | 3.69 | 1.76,  7.75 | 15 | 2.70 | 0.80,  9.12 | 1.26 | 0.51,  2.01 | 3.52 | 1.67,  7.43 |
| **Other** | 24 | 4.02 | 1.35, 11.98 | 1.07 | 0.43,  1.71 | 2.92 | 1.54,  5.51 | 24 | 3.75 | 1.26,  11.13 | 1.01 | 0.36,  1.65 | 2.73 | 1.43,  5.22 | 24 | 3.43 | 1.17,  10.06 | 0.96 | 0.30,  1.61 | 2.60 | 1.35,  5.00 |
|  |  |  |  |  |  |  |  |  |  |  |  |  |  |  |  |  |  |  |  |  |  |
| **Symptoms on presentation** | | | |  |  |  |  |  |  |  |  |  |  |  |  |  |  |  |  |  |  |
| **None reported** | 57 | Ref |  | Ref |  | Ref |  | 57 | Ref |  | Ref |  | Ref |  | 60 | Ref |  | Ref |  | Ref |  |
| **Depression &/or anxiety** | 159 | 3.52 | 1.64, 7.58 | 0.74 | 0.30,  1.19 | 2.10 | 1.34,  3.28 | 168 | 3.89 | 1.82,  8.29 | 0.83 | 0.38,  1.28 | 2.30 | 1.47,  3.60 | 177 | 3.47 | 1.69,  7.12 | 0.79 | 0.35,  1.23 | 2.21 | 1.42,  3.43 |
| **Loneliness** | 40 | 1.10 | 0.41, 2.92 | 0.08 | -0.50,  0.67 | 1.09 | 0.61,  1.94 | 41 | 1.21 | 0.46,  3.16 | 0.12 | -0.47,  0.71 | 1.13 | 0.63,  2.03 | 42 | 1.08 | 0.43,  2.72 | 0.18 | -0.40,  0.76 | 1.20 | 0.67,  2.15 |
| **Psychosis** | 10 | 0.81 | 0.16, 4.21 | -0.62 | -1.61,  0.37 | 0.54 | 0.20,  1.45 | 10 | 0.83 | 0.16,  4.28 | -0.58 | -1.59,  0.43 | 0.56 | 0.20,  1.53 | 10 | 0.76 | 0.15,  3.82 | -0.62 | -1.63,  0.40 | 0.54 | 0.19,  1.49 |
| **Suicidal thoughts/self, harm** | 61 | 2.80 | 1.16, 6.74 | 0.59 | 0.07,  1.11 | 1.81 | 1.08,  3.05 | 65 | 3.15 | 1.33,  7.44 | 0.72 | 0.20,  1.24 | 2.05 | 1.22,  3.46 | 67 | 2.79 | 1.22,  6.38 | 0.68 | 0.16,  1.19 | 1.97 | 1.18,  3.29 |
| **Other** | 137 | 1.97 | 0.91, 4.27 | 0.37 | -0.08,  0.81 | 1.45 | 0.93,  2.26 | 139 | 2.04 | 0.95,  4.38 | 0.40 | -0.05,  0.85 | 1.49 | 0.95,  2.34 | 143 | 1.84 | 0.89,  3.79 | 0.38 | -0.06,  0.83 | 1.47 | 0.94,  2.29 |
|  |  |  |  |  |  |  |  |  |  |  |  |  |  |  |  |  |  |  |  |  |  |
| **Financial concerns** | | | |  |  |  |  |  |  |  |  |  |  |  |  |  |  |  |  |  |  |
| **None** | 388 | Ref |  | Ref |  | Ref |  | 404 | Ref |  | Ref |  | Ref |  | 420 | Ref |  | Ref |  | Ref |  |
| **Yes** | 19 | 1.95 | 0.63, 3.45 | 0.38 | -0.21,  0.97 | 1.47 | 0.81,  2.64 | 19 | 1.79 | 0.69,  4.64 | 0.33 | -0.27,  0.93 | 1.39 | 0.77,  2.54 | 19 | 1.65 | 0.64,  4.25 | 0.28 | -0.32,  0.88 | 1.32 | 0.72,  2.42 |
| **(missing)** | 57 |  |  |  |  |  |  | 57 |  |  |  |  |  |  | 60 |  |  |  |  |  |  |
|  |  |  |  |  |  |  |  |  |  |  |  |  |  |  |  |  |  |  |  |  |  |
| **Lifestyle factors** | | | |  |  |  |  |  |  |  |  |  |  |  |  |  |  |  |  |  |  |
| **None** | 379 | Ref |  | Ref |  | Ref |  | 395 | Ref |  | Ref |  | Ref |  | 411 | Ref |  | Ref |  | Ref |  |
| **Yes** | 27 | 1.48 | 0.63, 3.45 | 0.34 | -0.17,  0.85 | 1.40 | 0.84,  2.34 | 27 | 1.38 | 0.59,  3.23 | 0.31 | -0.21,  0.83 | 1.37 | 0.81,  2.30 | 27 | 1.26 | 0.54,  2.92 | 0.26 | -0.26,  0.79 | 1.30 | 0.77,  2.20 |
| **(missing)** | 58 |  |  |  |  |  |  | 58 |  |  |  |  |  |  | 61 |  |  |  |  |  |  |
|  |  |  |  |  |  |  |  |  |  |  |  |  |  |  |  |  |  |  |  |  |  |
| **Physical health** | | | |  |  |  |  |  |  |  |  |  |  |  |  |  |  |  |  |  |  |
| **Not affected** | 365 | Ref |  | Ref |  | Ref |  | 380 | Ref |  | Ref |  | Ref |  | 392 | Ref |  | Ref |  | Ref |  |
| **Affected** | 40 | 1.13 | 0.53, 2.42 | 0.03 | -0.40,  0.46 | 1.03 | 0.67,  1.58 | 41 | 1.12 | 0.53,  2.36 | -0.01 | -0.44,  0.42 | 0.99 | 0.65,  1.53 | 45 | 1.27 | 0.63,  2.55 | 0.07 | -0.35,  0.48 | 1.07 | 0.71,  1.62 |
| **(missing)** | 59 |  |  |  |  |  |  | 59 |  |  |  |  |  |  | 62 |  |  |  |  |  |  |
|  |  |  |  |  |  |  |  |  |  |  |  |  |  |  |  |  |  |  |  |  |  |
| **Social support/isolation** | | | |  |  |  |  |  |  |  |  |  |  |  |  |  |  |  |  |  |  |
| **No concerns** | 311 | Ref |  | Ref |  | Ref |  | 324 | Ref |  | Ref |  | Ref |  | 336 | Ref |  | Ref |  | Ref |  |
| **Isolation** | 95 | 1.64 | 0.90, 2.99 | 0.35 | 0.00,  0.70 | 1.42 | 1.00,  2.01 | 98 | 1.60 | 0.88,  2.89 | 0.34 | -0.01,  0.68 | 1.40 | 0.99,  1.98 | 102 | 1.56 | 0.87,  2.82 | 0.30 | -0.05,  0.65 | 1.35 | 0.95,  1.91 |
| **(missing)** | 58 |  |  |  |  |  |  | 58 |  |  |  |  |  |  | 61 |  |  |  |  |  |  |
|  |  |  |  |  |  |  |  |  |  |  |  |  |  |  |  |  |  |  |  |  |  |
| **Presentation at the service** | | | | | | | | | | | | | | | | | | | | | |
| **First service contact** | | | |  |  |  |  |  |  |  |  |  |  |  |  |  |  |  |  |  |  |
| **Phone** | 405 | Ref |  | Ref |  | Ref |  | 420 | Ref |  | Ref |  | Ref |  | 438 | Ref |  | Ref |  | Ref |  |
| **Face to face** | 59 | 1.47 | 0.77, 2.79 | 0.15 | -0.24,  0.54 | 1.16 | 0.79,  1.72 | 60 | 1.34 | 0.71,  2.52 | 0.08 | -0.31,  0.48 | 1.09 | 0.73,  1.61 | 61 | 1.32 | 0.71,  2.46 | 0.06 | -0.33,  0.46 | 1.06 | 0.72,  1.58 |
