# Supplementary material for: Factors associated with repeat contact with an out-of-hours mental health crisis service: an observational study
Source: BMJ Public Health. 2025 Nov 10;3(2):e002924. doi: 10.1136/bmjph-2025-002924 (PMC12606496; doi:10.1136/bmjph-2025-002924)
Supplement: online supplemental figure 1 [file bmjph-3-2-s002.docx]

**Supplementary Figure 1. Directed Acyclic Graph for exposure variables for repeat attendance to Breathing Space services**


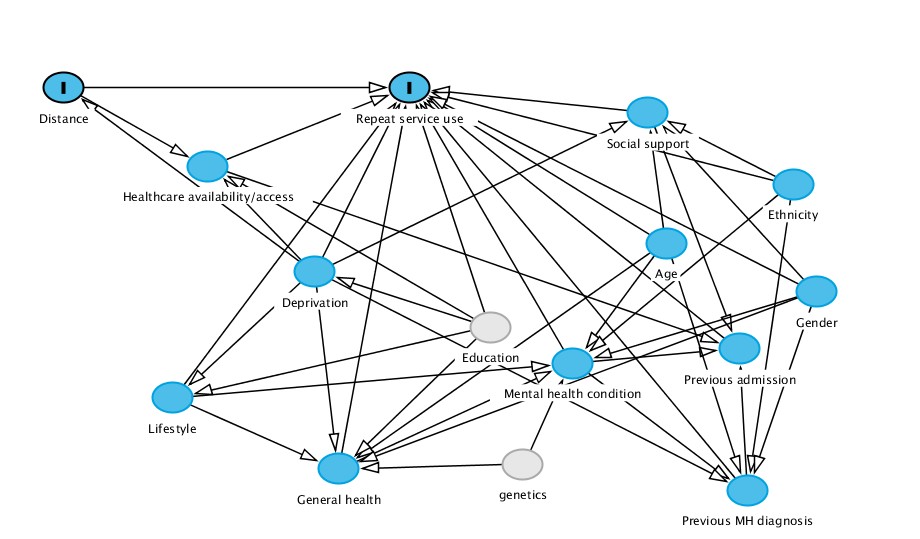


Blue = variable with potential to measure; Grey = variable that will not be able to measure therefore not included as an exposure variable.
